# Sodium-glucose cotransporter 2 inhibitors (SGLT2i) and cardiac arrhythmias: a systematic review and meta-analysis

**DOI:** 10.1186/s12933-021-01293-8

**Published:** 2021-05-07

**Authors:** Hang-Long Li, Gregory Y. H. Lip, Qi Feng, Yue Fei, Yi-Kei Tse, Mei-zhen Wu, Qing-wen Ren, Hung-Fat Tse, Bernard-M. Y. Cheung, Kai-Hang Yiu

**Affiliations:** 1 Division of Cardiology, Department of Medicine, The University of Hong Kong, Queen Mary Hospital, Room 1929B/K1931, Block K, Hong Kong, China; 2 Liverpool Centre for Cardiovascular Science, University of Liverpool and Liverpool Heart & Chest Hospital, Liverpool, UK; 3 Aalborg Thrombosis Research Unit, Department of Clinical Medicine, Aalborg University, Aalborg, Denmark; 4 Jockey Club School of Public Health and Primary Care, The Chinese University of Hong Kong, Hong Kong, China; 5 Division of Clinical Pharmacology, Department of Medicine, The University of Hong Kong, Queen Mary Hospital, Hong Kong, China; 6 Division of Cardiology, Department of Medicine, The University of Hong Kong Shenzhen Hospital, Shenzhen, China

**Keywords:** SGLT2 inhibitors, Arrhythmia, Atrial fibrillation

## Abstract

**Background:**

Cardiac arrhythmias are associated with poorer outcomes in patients with heart failure (HF), diabetes mellitus (DM), and chronic kidney disease (CKD). Previous studies have shown inconsistent conclusions regarding the association between sodium-glucose cotransporter 2 inhibitors (SGLT2i) and the risk of developing arrhythmias. This study aims to investigate the association of SGLT2i treatment with arrhythmia outcomes in clinical trials of patients with HF, DM, or CKD.

**Methods:**

MEDLINE, EMBASE, and ClinicalTrials.gov were searched from inception up to 27 August 2020. Randomized controlled trials that randomized patients with DM, CKD, or HF to SGLT2i or placebo were included. The outcomes of interest include atrial fibrillation (AF), embolic stroke, atrial flutter (AFL), AF/AFL, ventricular tachycardia (VT), and cardiac arrest. Relative risks (RRs) and 95% confidence intervals (CI) were pooled using a random-effects model.

**Results:**

Out of 4,532 citations, 22 trials with altogether 52,115 patients were included (mean age 63.2 years; 33,747 [64.8%] of participants were men). SGLT2i were associated with a lower risk of AF (RR 0.82, 95% CI 0.70–0.96), embolic stroke (RR 0.32, 95% CI 0.12–0.85), AF/AFL (RR 0.82, 95% CI 0.71–0.95), and VT (RR 0.73, 95% CI 0.53–0.99), while the risk reductions in AFL (RR 0.83, 95% CI 0.58–1.17) and cardiac arrest (RR 0.83, 95% CI 0.61–1.14) did not reach statistical significance. The associations appeared to be consistent across different baseline conditions (DM vs CKD vs HF; atherosclerotic cardiovascular disease [ASCVD] vs no ASCVD) and the SGLT2i used.

**Conclusions:**

SGLT2i reduced the risk of cardiac arrhythmias. Our study provides further evidence for recommending the use of SGLT2i in patients with DM, CKD, and HF. Further research is needed to fully elucidate the mechanism by which SGLT2i protect against arrhythmias.

**Supplementary Information:**

The online version contains supplementary material available at 10.1186/s12933-021-01293-8.

## Background

Diabetes mellitus (DM) and related comorbidities including heart failure (HF), obesity, hypertension, and chronic kidney disease (CKD) are closely linked to atrial fibrillation (AF) [1–5]. These conditions are associated with myocardial fibrosis and remodeling, neurohormonal activation, autonomic dysfunction, and electrical remodeling, predisposing to the development of AF and cardiac arrhythmias [2–6]. As AF and dysrhythmias are associated with a higher risk of adverse cardiovascular events and death, it is important to reduce the risk of cardiac arrhythmias in patients with HF, DM, and CKD [2, 4, 7–12].

Sodium-glucose cotransporter 2 inhibitors (SGLT2i) are antidiabetic medications which act by inhibiting the reabsorption of sodium and glucose in the proximal tubules of the kidney [13]. Commonly used SGLT2i include canagliflozin, dapagliflozin, and empagliflozin [13]. The cardioprotective effects of SGLT2i have been increasingly recognized in recent years: studies have shown that SGLT2i protected against atherosclerotic cardiovascular disease (ASCVD) and reduced HF hospitalization [13–15]. Furthermore, studies have shown that SGLT2i promoted weight loss and lowered blood pressure [13, 16]. In view of the wide spectrum of cardiovascular benefits, it has been hypothesized that SGLT2i may reduce the risk of AF and cardiac arrhythmias [17].

However, the associations between SGLT2i and AF remained inconsistent across previous studies. A recent secondary analysis of the DECLARE-TIMI 58 trial (Dapagliflozin Effect on Cardiovascular Events-Thrombolysis in Myocardial Infarction 58) found that dapagliflozin reduced the risk of AF and atrial flutter (AFL) by 19% in susceptible patients with DM, compared to placebo [17]. The reduction in AF/AFL events was consistent regardless of the presence of ASCVD, HF, and AF at baseline. However, one previous meta-analysis did not identify a significant association between SGLT2i and AF [18]. In the EMPA-REG OUTCOME trial (Empagliflozin Cardiovascular Outcome Event Trial in Type 2 Diabetes Mellitus Patients), the empagliflozin arm had a higher incidence of new-onset AF (2.3%) compared to the placebo arm (1.6%), though it was not an adjudicated outcome of the trial [19]. Recent real-world studies have also shown inconsistent conclusions: while SGLT2i were associated with a lower incidence of new-onset arrhythmias and AF, [20, 21] the CVD-REAL Nordic study [22] showed neutral association. Other types of arrhythmias, such as ventricular tachycardia (VT), as well as related conditions such as cardiac arrest, have been less well studied. Hence, the association between SGLT2i and arrhythmia outcomes remains uncertain.

Therefore, the objective of this systematic review and meta-analysis was to evaluate the effects of SGLT2i on common arrhythmia outcomes (AF, AFL, VT, and cardiac arrest) and related complications (embolic stroke) in patients with DM, CKD, and HF.

## Methods

This systematic review and meta-analysis was conducted and reported according to the Cochrane Handbook (Version 5.1.0) [23] and the PRISMA statement [24]. The PRISMA checklist is shown in Additional file 1: Table S1.

### Data sources and searches

Ovid MEDLINE, Ovid EMBASE, and ClinicalTrials.gov were searched for eligible studies through 27 August 2020. The search strategy is shown in Additional file 1: Table S2. Reviews articles and expert consensus statements were also manually searched for eligible studies.

### Study selection

We included RCTs that compared SGLT2i with placebo in adult patients (≥ 18 years) with type 2 DM, CKD, or HF and reported outcomes of interest as serious adverse events (SAEs). In order to ascertain the true anti-arrhythmic effects of SGLT2i, trials that randomized patients to combination therapy were excluded, and placebo was selected as a comparator. There were no restrictions on follow-up duration or the language of publication. The outcomes of interest include AF, embolic stroke, AFL, AF/AFL, VT, and cardiac arrest. Titles and abstracts were first screened to assess their potential eligibility, followed by full-text examination to determine final eligibility.

### Data extraction and quality assessment

The following information was extracted using a pre-specified data extraction form: bibliographic information (First author, year of publication), study information (trial name, ClinicalTrials.gov unique identifier, country, sample size), patients characteristics (age, proportion of male patients, baseline conditions and comorbidities (DM, CKD, HF, AF)), treatment information (regimen, dose, duration), and outcome data (number of events for each outcome). Since all outcomes of interest were binary, the 2*2 tables for each outcome were extracted. If multiple arms of the same drug at different doses were included in the same trial, the arms were combined into a single arm. This method is recommended in the Cochrane handbook [23] and was adopted in our previous meta-analysis [25]. When multiple studies of the same trial were found, the most updated publication/record was included. Data on outcomes of interest reported as SAEs on ClinicalTrials.gov were retrieved; data from the original trial publication or secondary analyses of the same trial were retrieved if no data could be extracted from ClinicalTrials.gov.

To assess methodological quality, the Cochrane Collaboration’s tool for assessing risk of bias was used [26]. Bias was assessed from seven domains: random sequence generation, allocation concealment, blinding of participants and personnel, blinding of outcome assessment, incomplete outcome data, selective reporting, and other biases. In each domain, bias was judged as high, low, or unclear. The overall risk of bias was judged as high if any domain was judged as high, as low if all domains were judged as low, or as unclear otherwise. The certainty of evidence was assessed using the Grading of Recommendations Assessment, Development, and Evaluation (GRADE) [27].

Study selection, data extraction, and quality assessment were conducted by two independent authors (HLL, and BMYC). Any disagreement was resolved by discussion until consensus was reached, or by consulting a third author (KHY).

### Data synthesis and analysis

The placebo arm was defined as the control in all analyses. Intention-to-treat analysis was employed. Relative risks (RR) and their 95% confidence intervals (CI) were pooled using a random-effects model with inverse variance weighting. RR < 1 would favor SGLT2i over placebo. Subgroup analysis was prespecified according to the baseline condition (HF vs DM vs CKD), presence of ASCVD at baseline, the SGLT2i agent used, and follow-up duration (≤ vs > median follow-up duration of all trials). Additional sensitivity analyses were performed by excluding studies with a high/unclear overall risk of bias, by excluding studies with a high/unclear bias in ‘Incomplete outcome data’, and by using odds ratio (OR) as the effect measure. To minimize the unbalanced representativeness of DM-only trials, a sensitivity analysis stratifying trials into DM versus other baseline conditions was performed. Statistical heterogeneity across studies was assessed by the Cochrane’s Q test and the I^2^ statistic. If substantial heterogeneity, as suggested by a p-value < 0.10 or I^2^ > 50%, was identified, meta-regression would be used to investigate potential sources of heterogeneity. Funnel plots were used for assessment of publication bias, and Egger’s test for asymmetry in funnel plot would only be performed if 10 or more studies were included [23]. Asymmetries in the funnel plots determined by visual assessment or a p-value for Egger’s test < 0.10 would suggest potential publication bias, and a trim-and-fill method was employed to adjust for potential bias. The statistical significance level was defined at 0.05 unless otherwise specified. Data analyses were performed using the “*meta*” package in R (version 3.6.3).

## Results

**Fig. 1:**
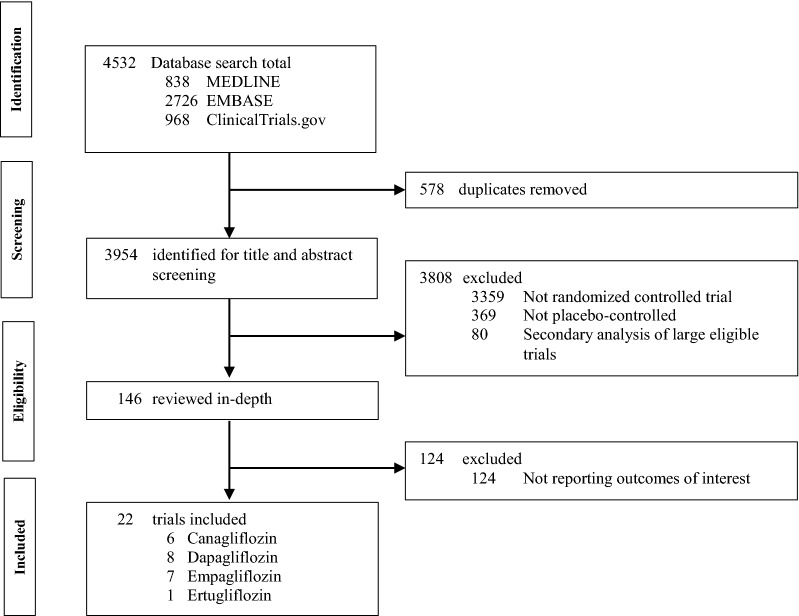
PRISMA flow diagram

Among the 4,532 citations identified by literature search, 22 trials [28–49] with altogether 52,115 patients (29,211 on SGLT2i and 22,904 on placebo) were included. The PRISMA flow diagram is shown in Fig. 1. The mean age was 63.2 years and 64.8% were male (Table 1). The median follow-up duration was 1.0 years (range 0.1 to 4.2). Seventeen trials enrolled patients with DM [28–44], three trials enrolled patients with DM and CKD [45–47], and two trials enrolled patients with HF [48, 49]. Overall, the percentages of the included participants with DM, CKD, and HF were 94.6%, 53.0% and 21.4%, respectively. The mean percentage of patients with a history of AF was 10.2% (range 5.5% to 70.9%). Eleven trials had a low risk of bias [32, 34–36, 39, 44–49], six trials had a high risk of bias [28, 29, 31, 33, 37, 43], and five trials had an unclear risk of bias [30, 38, 40–42] (Additional file 1: Table S3).

**Table 1:** Basic characteristics of eligible studies

Trial	Drug	Dose(s) analysed	Median follow-up duration (years)	ClinicalTrials.gov unique identifier	Total number of trial participants	Age (years), mean	Male, n (%)	Patients with a history of DM, n (%)	Patients with a history of CKD, n (%)	Patients with a history of HF, n (%)	Patients with a history of AF, n (%)
Bailey et al. [28]	Dapagliflozin	2.5 mg, 5 mg, 10 mg (once daily)	2.0	NCT00528879	546	53.9	292 (53.5%)	546 (100%)	NA	NA	NA
Bailey et al. [29]	Dapagliflozin	2.5 mg, 5 mg, 10 mg (once daily)	2.0	NCT00528372	558	52.2	276 (49.5%)	558 (100%)	NA	NA	NA
Bode et al. [30]	Canagliflozin	100 mg, 300 mg (once daily)	2.0	NCT01106651	714	63.6	396 (55.5%)	714 (100%)	NA	NA	NA
CANTATA-MSU [31]	Canagliflozin	100 mg, 300 mg (once daily)	1.0	NCT01106625	469	56.7	239 (51.0%)	469 (100%)	NA	NA	NA
CANVAS Program [32]	Canagliflozin	100 mg, 300 mg (once daily)	2.4	NCT01032629; NCT01989754	10,142	63.3	6509 (64.2%)	10,142 (100%)	1774 (17.5%)	1461 (14.4%)	612 (6.0%)
Cefalu et al. [33] 2015	Dapagliflozin	10 mg (once daily)	1.0	NCT01031680	922	62.9	624 (68.3%)	922 (100%)	NA	NA	NA
CREDENCE [47]	Canagliflozin	100 mg (once daily)	2.6	NCT02065791	4401	63.0	2907 (66.1%)	4401 (100%)	4401 (100%)	652 (15.8%)	NA
DAPA-HF [49]	Dapagliflozin	10 mg (once daily)	1.5	NCT03036124	4744	66.3	3635 (76.6%)	1983 (41.8%)	1926 (40.6%)	4744 (100%)	1818 (38.3%)
DECLARE-TIMI 58 [34]	Dapagliflozin	10 mg (once daily)	4.2	NCT01730534	17,160	63.9	10,738 (62.6%)	17,160 (100%)	8997 (52.4%)	1724 (10.0%)	1116 (6.5%)
EMPA-HEART CardioLink-6 [35]	Empagliflozin	10 mg (once daily)	0.5	NCT02998970	97	64.0^a^	90 (92.8%)	97 (100%)	2 (2.1%)	6 (6.2%)	NA
EMPA-REG OUTCOME [36]	Empagliflozin	10 mg, 25 mg (once daily)	3.1	NCT01131676	7020	63.1	5016 (71.5%)	7020 (100%)	5480 (78.1%)	706 (10.1%)	389 (5.5%)
EMPA-REG RENAL [45]	Empagliflozin	10 mg, 25 mg (once daily)	1.0	NCT01164501	738	63.9	430 (58.3%)	738 (100%)	738 (100%)	NA	NA
EMPA-RESPONSE-AHF [48]	Empagliflozin	10 mg (once daily)	0.1	NCT03200860	79	76^a^	53 (67.1%)	26 (32.9%)	NA	79 (100%)	56 (70.9%)
Inagaki et al. [37]	Canagliflozin	50 mg, 100 mg, 200 mg, 300 mg (once daily)	0.3	NCT01022112	383	57.4	261 (68.1%)	383 (100%)	29 (7.6%)	NA	NA
Kovacs et al. [40]	Empagliflozin	10 mg, 25 mg (once daily)	1.3	NCT01210001	498	54.5	241 (48.4%)	498 (100%)	NA	NA	NA
Leiter et al. [38]	Dapagliflozin	10 mg (once daily)	1.0	NCT01042977	962	63.8	644 (66.9%)	962 (100%)	NA	152 (15.8%)	NA
Mathieu et al. [41]	Dapagliflozin	10 mg (once daily)	0.5	NCT01646320	320	55.1	146 (45.6%)	320 (100%)	NA	NA	NA
Rosenstock et al. [39]	Empagliflozin	10 mg, 25 mg (once daily)	1.5	NCT01011868	494	58.8	276 (55.9%)	494 (100%)	NA	NA	NA
Softeland et al. [42]	Empagliflozin	10 mg, 25 mg (once daily)	0.5	NCT01734785	332	55.2	198 (59.6%)	332 (100%)	166 (50.0%)	NA	NA
VERTIS RENAL [46]	Ertugliflozin	5 mg, 15 mg (once daily)	1.0	NCT01986855	467	67.3	231 (49.5%)	467 (100%)	467 (100%)	NA	NA
Wilding et al. [43]	Dapagliflozin	2.5 mg, 5 mg, 10 mg (once daily)	1.0	NCT00673231	800	59.3	382 (47.8%)	800 (100%)	NA	NA	NA
Yale et al. [44]	Canagliflozin	100 mg, 300 mg (once daily)	1.0	NCT01064414	269	68.5	163 (60.6%)	269 (100%)	269 (100%)	NA	NA

*DM* diabetes mellitus, *CKD* chronic kidney disease, *HF* heart failure, *AF* atrial fibrillation, *NA* not available

^a^Median

**Fig. 2:**
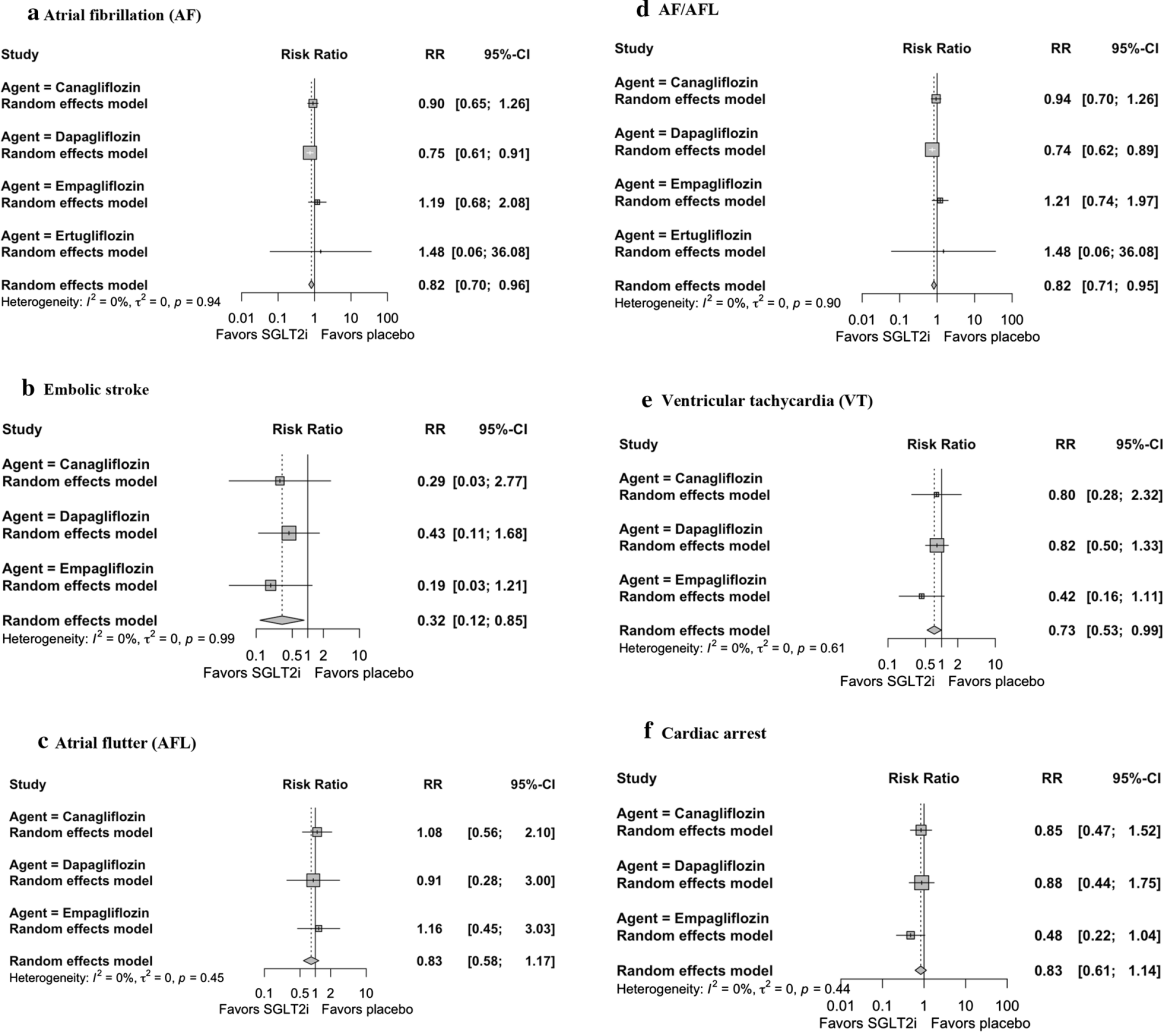
Forest plots of primary analysis

In total, 590 and 17 events of AF and embolic stroke were reported as SAEs, respectively. The RRs for AF ranged from 0.05 to 3.00, while RRs for embolic stroke ranged from 0.17 to 2.50. Overall, SGLT2i were associated with a 18% and 68% risk reduction in AF (RR 0.82, 95% CI 0.70–0.96) and embolic stroke (RR 0.32, 95% CI 0.12–0.85) compared to placebo (Fig. 2a, b). There was no significant heterogeneity across trials (p = 0.94 and p = 0.99 for AF and embolic stroke, respectively). In subgroup analysis according to the baseline condition (DM vs CKD vs HF), no significant between-subgroup heterogeneity was identified (p = 0.63 and p = 0.99 for AF and embolic stroke, respectively) (Table 2). Similarly, there were no significant heterogeneity in subgroup analysis according to the presence of ASCVD (p = 0.16 and p = 0.53 for AF and embolic stroke, respectively). In subgroup analysis according to the SGLT2i agent used, no significant between-subgroup heterogeneity was identified (p = 0.39 and p = 0.78 for AF and embolic stroke, respectively). There were no significant heterogeneity in subgroup analysis according to follow-up duration (p = 0.90 and p = 0.89 for AF and embolic stroke, respectively).

**Table 2:** Results of subgroup analysis

Outcome	Subgroup		Number of trials	Number of participants	RR (95% CI)	P_hetero_
AF	Overall		21	51,193	0.82 (0.70–0.96)	
	Baseline condition	DM	16	44,896	0.84 (0.71–1.00)	0.63
		CKD	3	1474	0.93 (0.18–4.68)	
		HF	2	4823	0.65 (0.40–1.07)	
	ASCVD	No ASCVD	18	43,114	0.79 (0.67–0.94)	0.16
		ASCVD present	3	8079	1.23 (0.69–2.19)	
	SGLT2i agent	Canagliflozin	6	16,378	0.90 (0.65–1.26)	0.39
		Dapagliflozin	7	25,090	0.75 (0.61–0.91)	
		Empagliflozin	7	9258	1.19 (0.68–2.08)	
		Ertugliflozin	1	467	1.48 (0.06–36.08)	
	Follow-up duration	≤ 1 year	11	4916	0.82 (0.69–0.97)	0.90
		> 1 year	10	42,677	0.82 (0.70–0.96)	
Embolic stroke	Overall		6	44,205	0.32 (0.12–0.85)	
	Baseline condition	DM	4	38,723	0.32 (0.10–1.03)	0.99
		CKD	1	4401	0.25 (0.01–6.21)	
		HF	1	4744	0.25 (0.03–3.20)	
	ASCVD	No ASCVD	5	37,185	0.37 (0.12–1.10)	0.53
		ASCVD present	1	7020	0.17 (0.02–1.59)	
	SGLT2i agent	Canagliflozin	2	14,543	0.29 (0.03–2.77)	0.78
		Dapagliflozin	3	21,904	0.43 (0.11–1.68)	
		Empagliflozin	4	7758	1.19 (0.03–1.21)	
	Follow-up duration	≤ 1 year	1	738	0.25 (0.01–6.21)	0.89
		> 1 year	5	43,467	0.32 (0.12–0.92)	
AFL	Overall		9	45,478	0.83 (0.58–1.17)	
	Baseline condition	DM	6	36,333	0.75 (0.52–1.09)	0.19
		CKD	1	4401	1.00 (0.14–7.08)	
		HF	1	4744	2.66 (0.71–10.03)	
	ASCVD	No ASCVD	6	37,439	0.91 (0.55–1.48)	0.90
		ASCVD present	3	8039	0.97 (0.36–2.66)	
	SGLT2i agent	Canagliflozin	2	14,543	1.08 (0.56–2.10)	0.95
		Dapagliflozin	3	22,826	0.91 (0.28–3.00)	
		Empagliflozin	4	8109	1.14 (0.45–3.03)	
	Follow-up duration	≤ 1 year	2	1019	0.33 (0.01–8.20)	0.56
		> 1 year	7	44,459	0.87 (0.59–1.29)	
AF/AFL	Overall		22	49,115	0.82 (0.71–0.95)	
	Baseline condition	DM	17	41,686	0.82 (0.70–0.97)	0.99
		CKD	3	5606	0.83 (0.46–1.51)	
		HF	2	4823	0.79 (0.51–1.24)	
	ASCVD	No ASCVD	18	40,114	0.79 (0.68–0.93)	0.16
		ASCVD present	4	9001	1.16 (0.70–1.91)	
	SGLT2i agent	Canagliflozin	6	16,378	0.94 (0.70–1.26)	0.20
		Dapagliflozin	8	26,012	0.74 (0.62–0.89)	
		Empagliflozin	7	9258	1.21 (0.74–1.97)	
		Ertugliflozin	1	467	1.48 (0.06–36.08)	
	Follow-up duration	≤ 1 year	12	5838	0.73 (0.31–1.71)	0.78
		> 1 year	10	42,677	0.82 (0.71–0.96)	
VT	Overall		7	43,963	0.73 (0.53–0.99)	
	Baseline condition	DM	4	34,739	0.90 (0.56–1.42)	0.50
		CKD	1	2501	0.50 (0.05–5.50)	
		HF	2	4823	0.62 (0.41–0.95)	
	ASCVD	No ASCVD	6	36,846	0.77 (0.56–1.06)	0.30
		ASCVD present	2	7117	0.44 (0.16–1.20)	
	SGLT2i agent	Canagliflozin	2	14,543	0.80 (0.28–2.32)	0.48
		Dapagliflozin	3	22,224	0.82 (0.50–1.33)	
		Empagliflozin	3	7196	0.42 (0.16–1.11)	
	Follow-up duration	≤ 1 year	3	496	0.98 (0.10–9.29)	0.79
		> 1 year	5	43,647	0.72 (0.53–0.99)	
Cardiac arrest	Overall		7	44,751	0.83 (0.61–1.14)	
	Baseline condition	DM	4	34,868	0.74 (0.42–1.30)	0.93
		CKD	2	5139	0.72 (0.25–2.08)	
		HF	1	4744	0.90 (0.37–2.21)	
	ASCVD	No ASCVD	6	37,731	0.92 (0.66–1.30)	0.12
		ASCVD present	1	7020	0.46 (0.20–1.03)	
	SGLT2i agent	Canagliflozin	2	14,543	0.85 (0.46–1.52)	0.44
		Dapagliflozin	3	22,450	0.88 (0.44–1.75)	
		Empagliflozin	2	7758	0.48 (0.22–1.04)	
	Follow-up duration	≤ 1 year	1	738	0.76 (0.05–12.13)	0.97
		> 1 year	6	44,013	0.81 (0.56–1.16)	

*AF* atrial fibrillation, *AFL* atrial flutter, *VT* ventricular tachycardia, *RR* risk ratio, *95% CI* 95% confidence interval, *P*_*hetero*_ P-value for between-subgroup heterogeneity, *DM* diabetes mellitus, *CKD* chronic kidney disease, *HF* heart failure, *ASCVD* atherosclerotic cardiovascular disease

A total of 135 events of AFL were reported as SAEs. The RRs for AFL ranged from 0.33 to 2.66. Overall, SGLT2i did not significantly influence the risk of AFL (RR 0.83, 95% CI 0.68–1.17) compared to placebo (Fig. 2c). There was no significant heterogeneity across trials (p = 0.45). In subgroup analysis according to baseline condition, according to the presence of ASCVD, according to the SGLT2i agent used, and according to follow-up duration, no significant between-subgroup heterogeneity was identified (p = 0.19, p = 0.90, p = 0.95, and p = 0.56, respectively) (Table 2).

When AF and AFL are combined as a composite endpoint, SGLT2i are associated with an 18% risk reduction in AF/AFL (RR 0.82, 95% CI 0.71–0.95) (Fig. 2d). There was no significant heterogeneity across trials (p = 0.90). Subgroup analysis stratifying studies according to baseline condition, presence of ASCVD, SGLT2i agent, and follow-up duration did not identify a significant between-subgroup heterogeneity (p = 0.99, p = 0.16, p = 0.20, and p = 0.78, respectively) (Table 2).

A total of 163 events of VT were reported as SAEs. The RRs for VT ranged from 0.33 to 3.00. Overall, SGLT2i were associated with a 27% risk reduction in VT compared to placebo (RR 0.73, 95% CI 0.53–0.99) (Fig. 2e). There was no significant heterogeneity across trials (p = 0.61). Subgroup analysis stratifying studies according to baseline condition, presence of ASCVD, SGLT2i agent, and follow-up duration did not identify a significant between-subgroup heterogeneity (p = 0.50, p = 0.30, p = 0.48, and p = 0.79, respectively) (Table 2).

A total of 157 cardiac arrest events were reported as SAEs. The RRs for cardiac arrest ranged from 0.07 to 1.06. Overall, SGLT2i did not significantly influence the risk of cardiac arrest (RR 0.83, 95% CI 0.61–1.14) (Fig. 2f). There was no significant heterogeneity across trials (p = 0.44). Subgroup analysis according to baseline condition, presence of ASCVD, SGLT2i agent, and follow-up duration did not identify a significant between-subgroup heterogeneity (p = 0.93, p = 0.12, p = 0.44, and p = 0.97, respectively) (Table 2).

Sensitivity analyses excluding studies with a high/unclear overall risk of bias, excluding studies with a high/unclear risk of bias in Incomplete outcome data, and using OR as an effect measure yielded largely consistent results (Additional file 1: Table S4(A)). In the sensitivity analysis stratifying trials according to DM versus other baseline conditions (CKD or HF), there were no significant subgroup differences (Additional file 1: Table S4(B)). Symmetry was observed in the funnel plots for AF, embolic stroke, AF/AFL, VT, and cardiac arrest, but not for AFL (Additional file 1: Fig. S1). Egger’s test for AF and AF/AFL did not reveal significant asymmetry, whereas Egger’s test was not performed for embolic stroke, AFL, VT, and cardiac arrest since the number of studies was below 10. Trim-and-fill method generated an overall RR of 0.77 (95% CI 0.52–1.14) for AFL. The GRADE assessment for each outcome was shown in Additional file 1: Table S5. The certainty of evidence for AF, embolic stroke, AF/AFL, and VT were graded as high, whereas the certainty for AFL and cardiac arrest were graded as moderate due to imprecision (as the 95% of the relative risk was sufficiently wide that the estimate could include appreciable benefit/harm of the use of SGLT2i, with 0.75 and 1.25 taken as thresholds).

## Discussion

In this systematic review and meta-analysis of 22 trials with 52,115 patients with DM, CKD, or HF susceptible to developing arrhythmias, we found that SGLT2i treatment might be associated with a lower risk of AF, embolic stroke, AF/AFL, and VT, compared to placebo. The associations appeared to be consistent across all baseline conditions (HF vs DM vs CKD; ASCVD vs no ASCVD), all SGLT2i subgroups, and across short vs long follow-up duration. Although no significant associations were observed for AFL and cardiac arrest, the point estimates appeared to be consistent with that of AF. These findings are consistent with recent reports suggesting that SGLT2i reduced the risk of arrhythmias [17, 20].

To the authors’ knowledge, this is the largest and most comprehensive systematic review and meta-analysis that addresses the association between SGLT2i and arrhythmia outcomes.

A previous meta-analysis did not find a significant association between SGLT2i treatment and AF (OR 0.61, 95% CI 0.31–1.19) [18]. As the number of participants (52,115 vs 10,512) and events (590 vs 30) are much larger in our meta-analysis, the association we identified, which suggests a significant risk reduction in AF with SGLT2i treatment, is more likely to be robust. In the EMPA-REG OUTCOME trial, although the incidence of new-onset AF appeared to be higher in the SGLT2i group (2.3%) than in the placebo group (1.6%), the difference did not reach statistical significance [19]. Furthermore, no significant difference between SGLT2i subgroups was identified in our meta-analysis. Previous meta-analyses have identified consistent risk reductions in adverse cardiovascular/renal events across different SGLT2i agents, and empagliflozin is likely to exhibit similar cardio- and reno-protective properties [14, 50]. Nevertheless, a recent real-world cohort study of patients with DM found that empagliflozin resulted in poorer outcome in reduction of HF compared to dapagliflozin [51]. Therefore, larger studies evaluating the effects of empagliflozin on AF are required to confirm the association.

A previous meta-analysis found that SGLT2i did not significantly influence the risk of stroke [52]. However, embolic stroke was not specifically studied as an outcome. No previous studies have evaluated the association between SGLT2i and embolic stroke, and the current meta-analysis is the first to report the protective effect of SGLT2i on embolic stroke. Such association might be attributed to the risk reduction in AF. Nevertheless, owing to the low number of events reported in the included trials, larger studies evaluating the association between SGLT2i and embolic stroke are needed to confirm our findings.

Although our meta-analysis failed to identify a significant risk reduction in AFL, a statistically significant risk reduction was identified when AF and AFL were evaluated as a composite outcome. A similar risk reduction in AF/AFL events (RR 0.81, 95% CI 0.67–0.98) was identified in the secondary analysis of the DECLARE-TIMI 58 trial [17]. The low number of AFL events observed in the included trials in our meta-analysis might have contributed to a wide confidence interval, hence a marginally significant association. Larger RCTs powered to detect differences in AFL are required to confirm these findings. Nevertheless, AF and AFL have similar clinical significance and consequences, [53] and the conjoint analysis of AF/AFL, which shows a significant risk reduction, provides more robust results while obviating possible publication bias for AFL.

A previous meta-analysis by Li et al. identified a 24% risk reduction in AF/AFL with SGLT2i treatment, [54] as compared to 18% and 17% risk reduction in AF and AFL, respectively, in our meta-analysis. The inconsistency could be explained by the significant methodological differences. In addition to trials of DM patients, our meta-analysis also included trials of HF and CKD patients, resulting in a significantly higher number of trials (22 vs 16) and patients (52,115 vs 38,335) included. Therefore, our observations are likely to be more robust and accurate. More importantly, as the use of SGLT2i has greatly expanded from selected DM patients only to patients with DM, CKD, and cardiovascular diseases, [55] our findings are applicable to patients with a much broader spectrum of comorbidities. Furthermore, the meta-analysis by Li et al. only evaluated AF/AFL as an arrhythmia outcome, as compared to four additional outcomes (AF, embolic stroke, AFL, VT, and cardiac arrest) evaluated in our meta-analysis, highlighting the comprehensiveness of the present study. Our meta-analysis provides a more holistic evaluation of how SGLT2i reduced the risk of arrhythmias.

The association between SGLT2i, VT, and cardiac arrest has been less well studied. To our knowledge, this meta-analysis is the first study to address this research question. SGLT2i treatment was associated with a 28% risk reduction for VT. In the recent EMBODY trial, [56] improvements in indicators of cardiac sympathetic/parasympathetic nerve activity, which are related to the risk of ventricular tachyarrhythmias, were greater in the empagliflozin group compared to the placebo group. Only the empagliflozin group achieved a significant intra-group improvement. Taking these findings together, it is likely that SGLT2i may exert a protecting effect against VT. Meanwhile, six out of seven studies reported an RR of < 1.0 for cardiac arrest, and there was a 17% risk reduction in developing cardiac arrest. The marginally significant association could be attributed to a low number of events, and larger prospective studies are warranted to confirm the association.

It is being increasingly recognized that HF, DM, and CKD are associated with AF and cardiac arrhythmias [1–4, 9]. The presence of AF is associated with a higher risk of adverse cardiovascular events, cardiovascular mortality, and all-cause mortality in individuals with HF, CKD, and DM [2, 4, 7–9]. Similar associations were observed for AFL and VT: HF and DM may predispose to the development of AFL and VT, which are in turn associated with higher mortality [10–12]. Therefore, it is of critical importance to reduce the risk of arrhythmias in patients with HF, DM, and CKD.

The pathophysiological pathways linking DM, CKD, and HF with the development of AF and arrhythmias are complex and multifactorial [2, 3, 6]. The presence of DM has been implicated to explain the coexistence of CKD, HF, and AF [3, 4]. Compared to the general population, individuals with DM and stage 5 CKD have a threefold increased risk to develop AF, [5] in which comorbid DM increases the risk of bleeding in patients with AF [57]. DM has also been found to increase the risk of suboptimal response to cardiac resynchronization therapy with defibrillator in patients with HF [58]. Furthermore, multiple signaling pathways contribute to remodeling and arrhythmogenic properties in HF, increasing the risk of developing ventricular tachyarrhythmias [9, 59]. There are also several possible mechanisms through which SGLT2i reduce the risk of arrhythmias. By promoting osmotic diuresis and natriuresis, SGLT2i alleviate cardiac workload and improve left ventricular function [60]. SGLT2i may also reduce arrhythmia by modulating neurohormonal pathways, which, in DM, CKD, and HF, are activated and play important roles in the deterioration of these conditions [61, 62]. By optimizing hemodynamic status, SGLT2i reduce fluid overload, which is associated with cardiac structural abnormalities, hence predisposition to arrhythmia, in DM and CKD [63]. SGLT2i are also effective in blood pressure and glycemic control, both of which are implicated in cardiac arrhythmogenesis [64]. Furthermore, by inhibiting the myocardial sodium-hydrogen exchanger (Na^+^/H^+^ exchanger), which is upregulated in HF, SGLT2i lead to improvement in mitochondrial dysfunction and reduction in oxidative stress, thus reducing the risk of arrhythmias [65, 66]. Apart from improving mitochondrial function, [67] SGLT2i have also been found to alleviate atrial remodeling, an important process implicated in atrial arrhythmogenesis [68]. SGLT2i may also reduce electrical instability by ensuring a sufficient energy supply [69–71]. Other possible mechanisms are outlined in Additional file 1: Fig. S2.

There are several limitations in this meta-analysis. First, arrhythmia outcomes were not the pre-specified outcomes of the included trials, and there might be ascertainment bias. The outcomes were not adjudicated and might lead to inaccuracies and incompleteness of data. Nevertheless, in sensitivity analyses where studies with high/unclear overall risk of bias and studies with high/unclear risk of bias in ‘Incomplete outcome data’ were excluded, largely consistent associations were observed. Nevertheless, the approach of using adverse events as outcomes has been used in previous studies [21, 54]. Nonetheless, further randomized trials with well-defined and adjudicated arrhythmia outcomes are required to confirm the associations reported in the current study. Second, the included trials were underpowered to detect differences in arrhythmia outcomes. Future trials designed with arrhythmias as the primary outcomes are warranted. Third, outcomes on arrhythmia-related mortality were not included. These outcomes are clinically more important but were not reported in the identified trials. AF confers higher mortality in patients with DM, CKD, and HF, and further studies examining the effects of SGLT2i on arrhythmia-related death are urgently needed. Fourth, as patient-level data were not available and not all trials reported the baseline prevalence of DM/CKD/HF, trials could not be grouped according to a combination of disease processes, for instance, DM + CKD + HF. Therefore, further studies are required to evaluate whether SGLT2 inhibitors could reduce incidences of arrhythmia in patients with multiple comorbidities. Fifth, as no data on the number of events specific to baseline comorbidity, for instance, the number of AF events in those with pre-existing HF vs those without, were available from the trial, the results of subgroup analyses should be interpreted with caution, and further studies are warranted to confirm that the associations between SGLT2i and arrhythmias remain significant regardless of the presence of baseline comorbidity. Sixth, patient-level data on pre-existing AF and the use of anti-arrhythmic medications were not available from most trials.

## Conclusions

This meta-analysis found that SGLT2i reduced the risk of AF and VT. Our study provides further robust evidence for recommending the use of SGLT2i in patients with DM, CKD, and HF to reduce related cardiac complications and comorbidities. However, the mechanisms by which SGLT2i protects against arrhythmias are complex and further research is warranted.

## Supplementary Information

**Additional file 1:Figure S1. **Funnel plots and results of Egger’s test for asymmetry. **Figure S2**. Proposed mechanisms through which SGLT2 inhibitors reduce risk of arrhythmias. **Table S1.** PRISMA checklist. **Table S2.** Search strategy. **Table S3.** Risk of bias assessment. **Table S4.** Results of sensitivity analyses. **Table S5.** GRADE assessment.

## Data Availability

The review has not been registered; The dataset(s) supporting the conclusions of this article is(are) included within the article.

## Abbreviations

AF: Atrial fibrillation
AFL: Atrial flutter
ASCVD: Atherosclerotic cardiovascular disease
CKD: Chronic kidney disease
DM: Diabetes mellitus
HF: Heart failure
OR: Odds ratio
RR: Relative risk
SAEs: Serious adverse events
SGLT2i: Sodium-glucose cotransporter 2 inhibitors
VT: Ventricular tachycardia
