# Correction to: Sodium–glucose cotransporter 2 inhibitors (SGLT2i) and cardiac arrhythmias: a systematic review and meta‑analysis

**DOI:** 10.1186/s12933-021-01371-x

**Published:** 2021-09-04

**Authors:** Hang-Long Li, Gregory Y. H. Lip, Qi Feng, Yue Fei, Yi-Kei Tse, Mei-zhen Wu, Qing-wen Ren, Hung-Fat Tse, Bernard-M. Y. Cheung, Kai-Hang Yiu

**Affiliations:** 1 Division of Cardiology, Department of Medicine, The University of Hong Kong, Queen Mary Hospital, Room 1929B/K1931, Block K, Hong Kong, China; 2 Liverpool Centre for Cardiovascular Science, University of Liverpool and Liverpool Heart & Chest Hospital, Liverpool, UK; 3 Aalborg Thrombosis Research Unit, Department of Clinical Medicine, Aalborg University, Aalborg, Denmark; 4 Jockey Club School of Public Health and Primary Care, The Chinese University of Hong Kong, Hong Kong, China; 5 Division of Clinical Pharmacology, Department of Medicine, The University of Hong Kong, Queen Mary Hospital, Hong Kong, China; 6 Division of Cardiology, Department of Medicine, The University of Hong Kong Shenzhen Hospital, Shenzhen, China

## Correction to: Cardiovasc Diabetol (2021) 20:100 10.1186/s12933-021-01293-8

Following publication of the original article [1], the authors noticed an error in the second author’s name.

The name of the second author, "Gregory Y. H. Lip", was incorrectly written as "Gregory-Y H Lip". This has been corrected with this erratum.

The original article [1] has been corrected.
