# Supplementary material for: Sodium-glucose cotransporter 2 inhibitors (SGLT2i) and cardiac arrhythmias: a systematic review and meta-analysis
Source: Cardiovasc Diabetol. 2021 May 7;20:100. doi: 10.1186/s12933-021-01293-8 (PMC8106208; doi:10.1186/s12933-021-01293-8)
Supplement: Supplementary file 1 — Additional file 1:Figure S1. Funnel plots and results of Egger’s test for asymmetry. Figure S2. Proposed mechanisms through which SGLT2 inhibitors reduce risk of arrhythmias. Table S1. PRISMA checklist. Table S2. Search strategy. Table S3. Risk of bias assessment. Table S4. Results of sensitivity analyses. Table S5. GRADE assessment. [file 12933_2021_1293_MOESM1_ESM.pdf]

Appendix Figure 1. Funnel plots and results of Egger's test for asymmetry.

(A) Atrial fibrillation (AF)

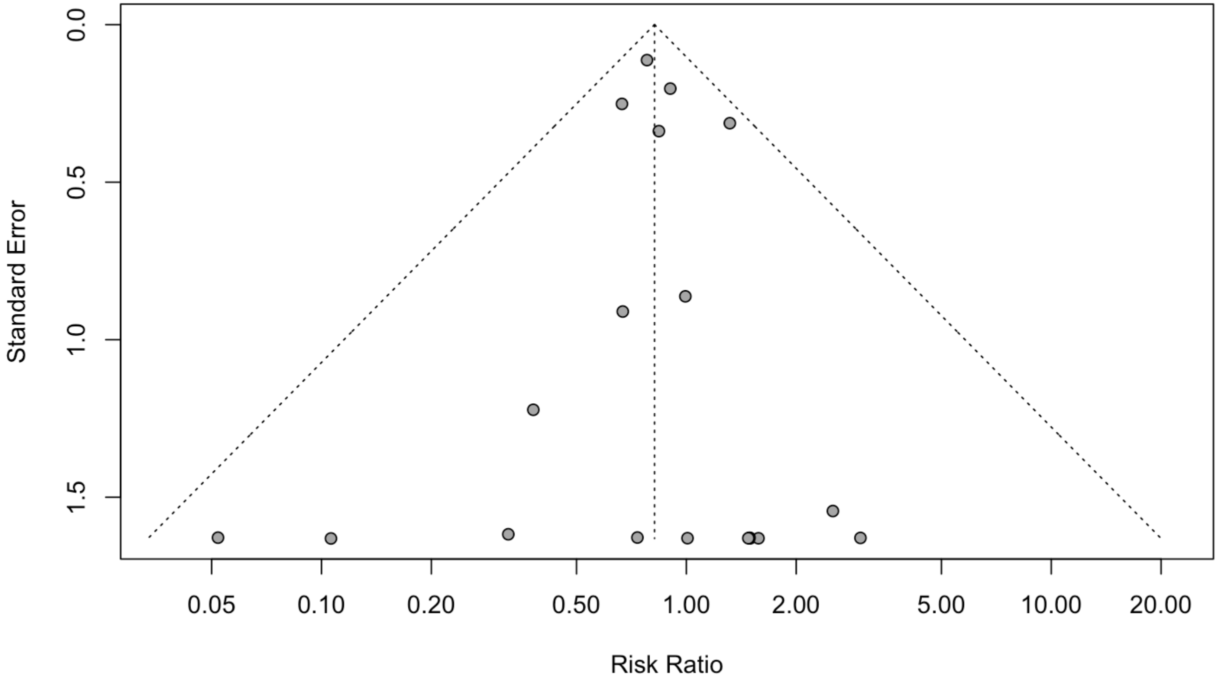

P-value for Egger's test: 0.9477

(B) Embolic stroke

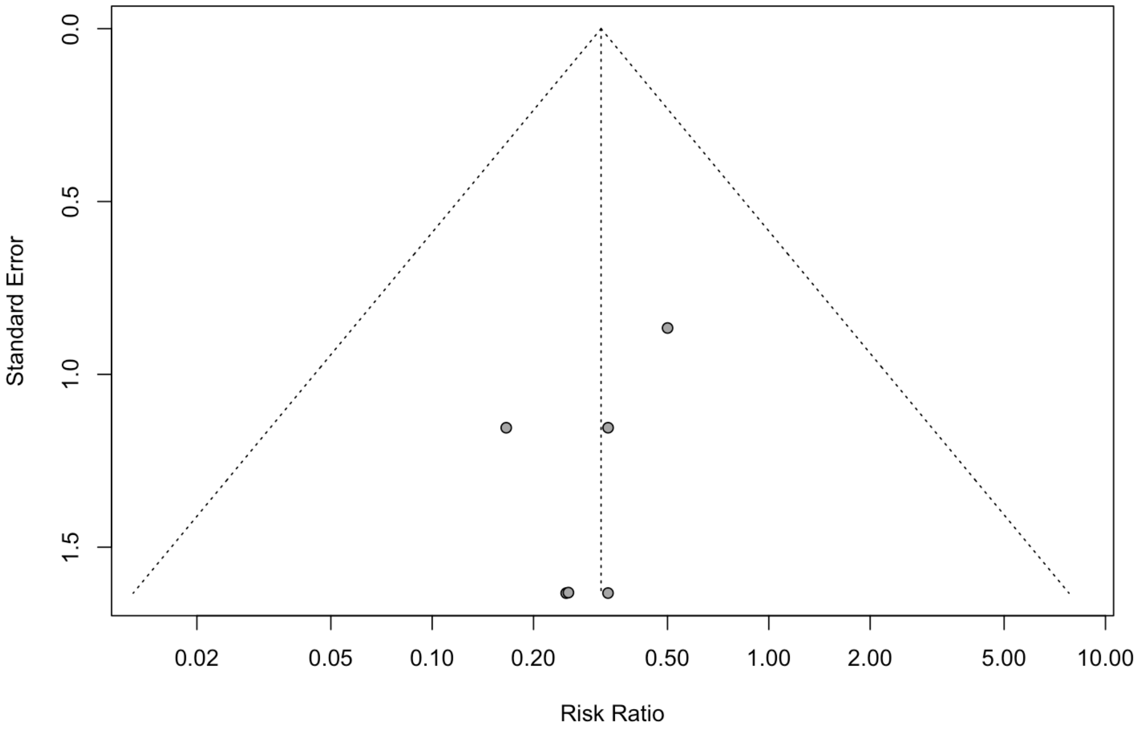

(C) Atrial flutter (AFL)

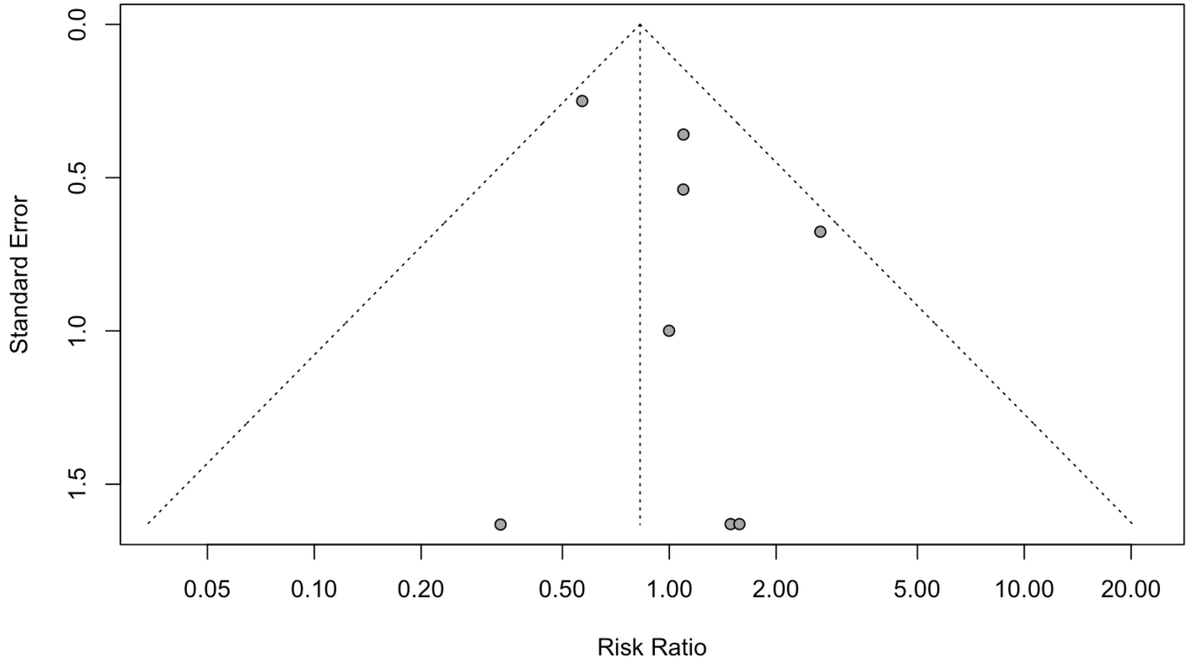

(D) AF/AFL

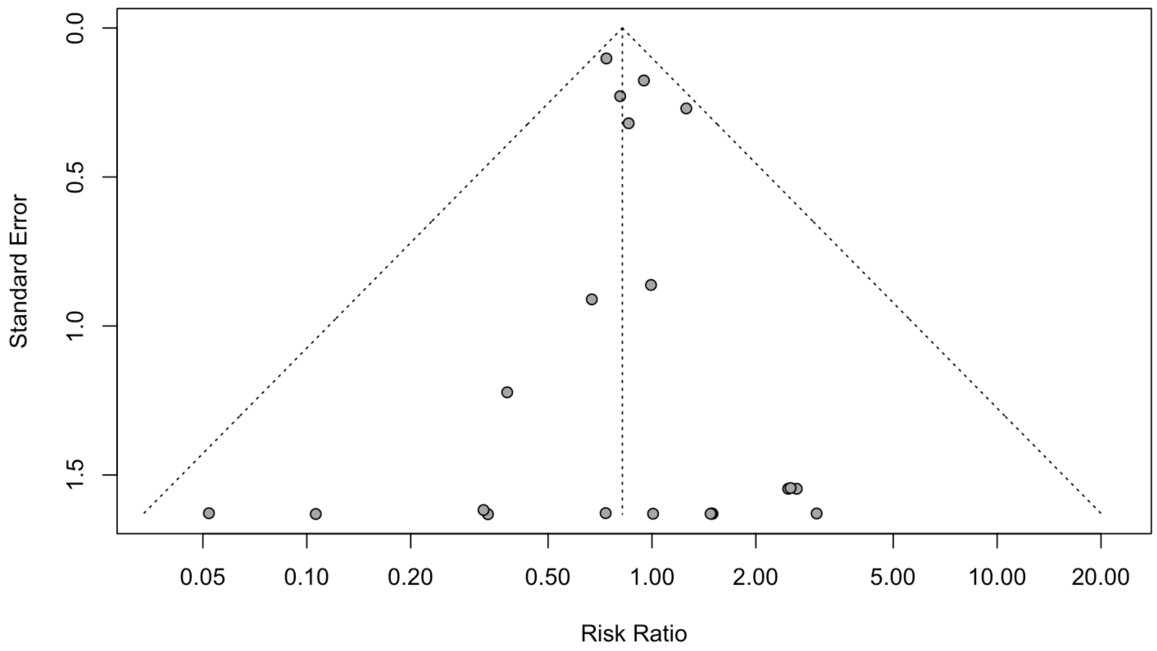

P-value for Egger's test: 0.7716

(E) Ventricular tachycardia (VT)

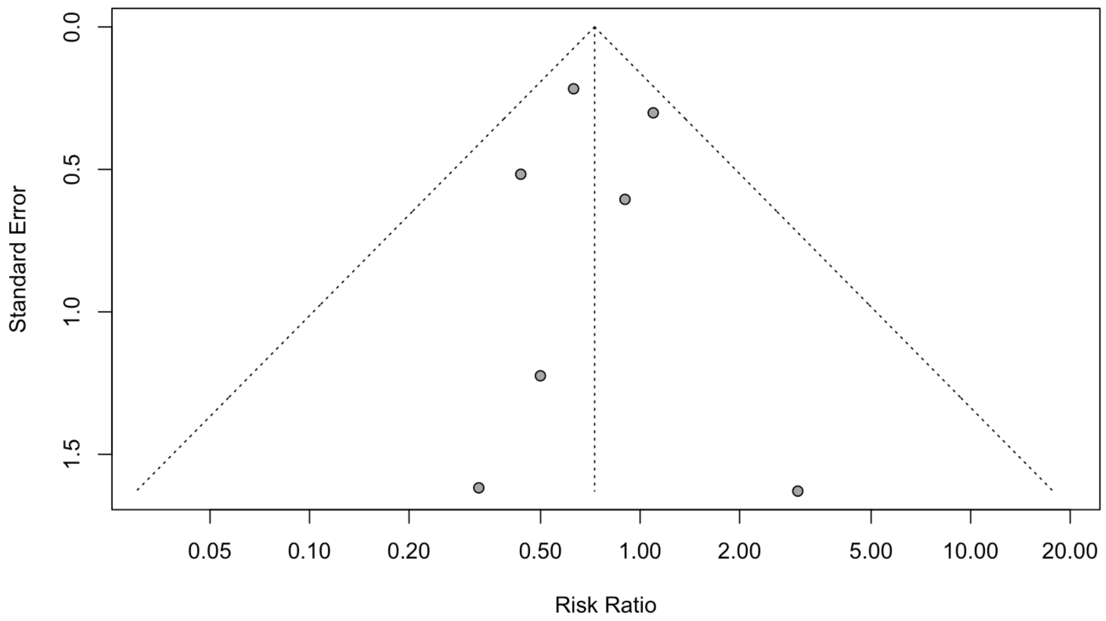

(F) Cardiac arrest

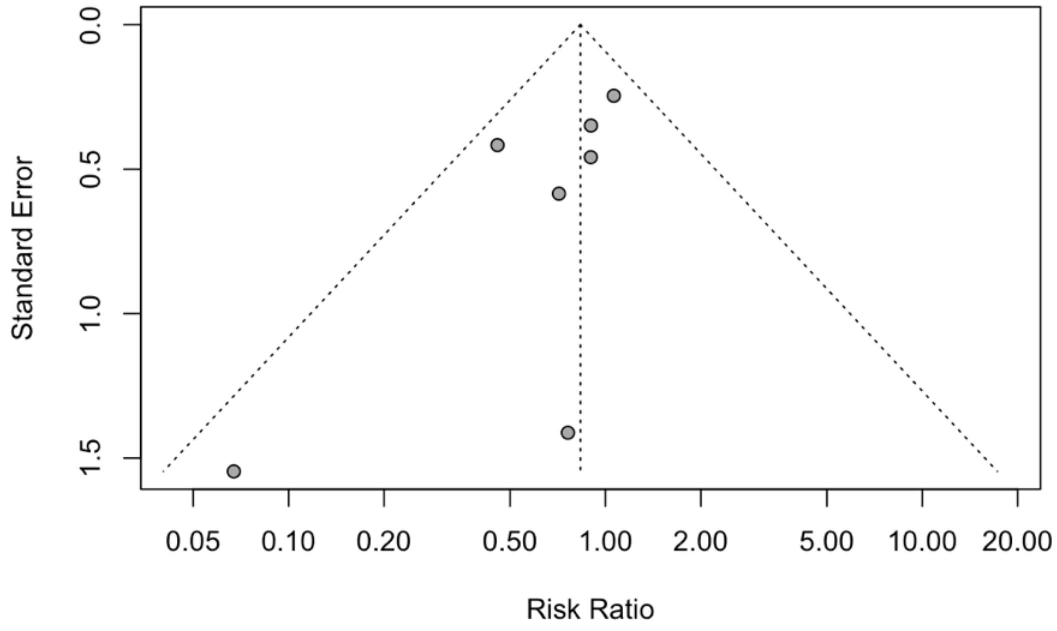

Appendix Figure 2. Proposed mechanisms through which SGLT2 inhibitors reduce risk of arrhythmias.

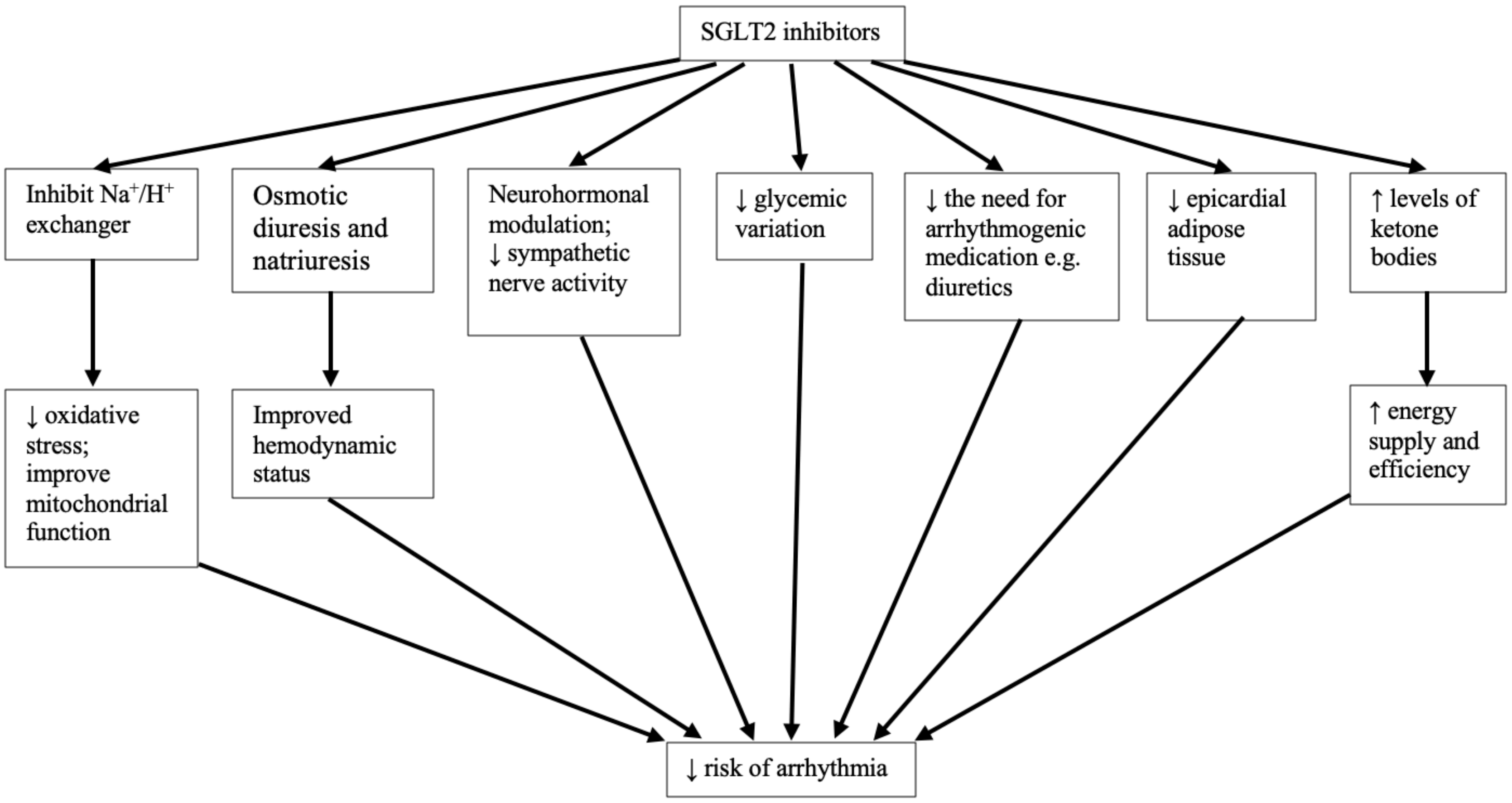

PRISMA 2020 Checklist

Appendix Table 1. PRISMA checklist.

Section and Topic	Item #	Checklist item	Page/Location where item is reported
**TITLE**			
Title	1	Identify the report as a systematic review.	1
**ABSTRACT**			
Abstract	2	See the PRISMA 2020 for Abstracts checklist.	3-4
**INTRODUCTION**			
Rationale	3	Describe the rationale for the review in the context of existing knowledge.	5-6
Objectives	4	Provide an explicit statement of the objective(s) or question(s) the review addresses.	6
**METHODS**			
Eligibility criteria	5	Specify the inclusion and exclusion criteria for the review and how studies were grouped for the syntheses.	7
Information sources	6	Specify all databases, registers, websites, organisations, reference lists and other sources searched or consulted to identify studies. Specify the date when each source was last searched or consulted.	7
Search strategy	7	Present the full search strategies for all databases, registers and websites, including any filters and limits used.	Appendix Table 2
Selection process	8	Specify the methods used to decide whether a study met the inclusion criteria of the review, including how many reviewers screened each record and each report retrieved, whether they worked independently, and if applicable, details of automation tools used in the process.	7-8
Data collection process	9	Specify the methods used to collect data from reports, including how many reviewers collected data from each report, whether they worked independently, any processes for obtaining or confirming data from study investigators, and if applicable, details of automation tools used in the process.	7-8
Data items	10a	List and define all outcomes for which data were sought. Specify whether all results that were compatible with each outcome domain in each study were sought (e.g. for all measures, time points, analyses), and if not, the methods used to decide which results to collect.	7-8
	10b	List and define all other variables for which data were sought (e.g. participant and intervention characteristics, funding sources). Describe any assumptions made about any missing or unclear information.	7-9
Study risk of bias assessment	11	Specify the methods used to assess risk of bias in the included studies, including details of the tool(s) used, how many reviewers assessed each study and whether they worked independently, and if applicable, details of automation tools used in the process.	8
Effect measures	12	Specify for each outcome the effect measure(s) (e.g. risk ratio, mean difference) used in the synthesis or presentation of results.	8-9
Synthesis methods	13a	Describe the processes used to decide which studies were eligible for each synthesis (e.g. tabulating the study intervention characteristics and comparing against the planned groups for each synthesis (item #5)).	7
	13b	Describe any methods required to prepare the data for presentation or synthesis, such as handling of missing summary statistics, or data conversions.	8-9
	13c	Describe any methods used to tabulate or visually display results of individual studies and syntheses.	9
	13d	Describe any methods used to synthesize results and provide a rationale for the choice(s). If meta-analysis was performed, describe the model(s), method(s) to identify the presence and extent of statistical heterogeneity, and software package(s) used.	8-9
	13e	Describe any methods used to explore possible causes of heterogeneity among study results (e.g. subgroup analysis, meta-regression).	9
	13f	Describe any sensitivity analyses conducted to assess robustness of the synthesized results.	9
Reporting bias assessment	14	Describe any methods used to assess risk of bias due to missing results in a synthesis (arising from reporting biases).	9

PRISMA 2020 Checklist

Section and Topic	Item #	Checklist item	Page/Location where item is reported
Certainty assessment	15	Describe any methods used to assess certainty (or confidence) in the body of evidence for an outcome.	
**RESULTS**			
Study selection	16a	Describe the results of the search and selection process, from the number of records identified in the search to the number of studies included in the review, ideally using a flow diagram.	10; Figure 1
	16b	Cite studies that might appear to meet the inclusion criteria, but which were excluded, and explain why they were excluded.	10
Study characteristics	17	Cite each included study and present its characteristics.	10; Table 1
Risk of bias in studies	18	Present assessments of risk of bias for each included study.	10; Appendix Table 3
Results of individual studies	19	For all outcomes, present, for each study: (a) summary statistics for each group (where appropriate) and (b) an effect estimate and its precision (e.g. confidence/credible interval), ideally using structured tables or plots.	10-12; Figure 2
Results of syntheses	20a	For each synthesis, briefly summarise the characteristics and risk of bias among contributing studies.	12
	20b	Present results of all statistical syntheses conducted. If meta-analysis was done, present for each the summary estimate and its precision (e.g. confidence/credible interval) and measures of statistical heterogeneity. If comparing groups, describe the direction of the effect.	10-12
	20c	Present results of all investigations of possible causes of heterogeneity among study results.	10-12
	20d	Present results of all sensitivity analyses conducted to assess the robustness of the synthesized results.	12; Appendix Table 4
Reporting biases	21	Present assessments of risk of bias due to missing results (arising from reporting biases) for each synthesis assessed.	10-12
Certainty of evidence	22	Present assessments of certainty (or confidence) in the body of evidence for each outcome assessed.	
**DISCUSSION**			
Discussion	23a	Provide a general interpretation of the results in the context of other evidence.	13-17
	23b	Discuss any limitations of the evidence included in the review.	16-17
	23c	Discuss any limitations of the review processes used.	16-17
	23d	Discuss implications of the results for practice, policy, and future research.	13-17
**OTHER INFORMATION**			
Registration and protocol	24a	Provide registration information for the review, including register name and registration number, or state that the review was not registered.	18
	24b	Indicate where the review protocol can be accessed, or state that a protocol was not prepared.	18
	24c	Describe and explain any amendments to information provided at registration or in the protocol.	18
Support	25	Describe sources of financial or non-financial support for the review, and the role of the funders or sponsors in the review.	19
Competing interests	26	Declare any competing interests of review authors.	18
Availability of data, code and other materials	27	Report which of the following are publicly available and where they can be found: template data collection forms; data extracted from included studies; data used for all analyses; analytic code; any other materials used in the review.	18

PRISMA 2020 Checklist

From: Page MJ, McKenzie JE, Bossuyt PM, Boutron I, Hoffmann TC, Mulrow CD, et al. The PRISMA 2020 statement: an updated guideline for reporting systematic reviews. BMJ 2021;372:n71. doi: 10.1136/bmj.n71

For more information, visit: http://www.prisma-statement.org/

Appendix Table 2. Search strategy.

**Intervention**	#1	‘Sodium-Glucose Transporter 2’ OR ‘Sodium-Glucose Transporter 2 inhibitor’ OR ‘SGLT2 inhibitor’ OR ‘*gliflozin’
	#2	‘canagliflozin’ OR ‘invokana’ OR ‘sulisent’ OR ‘TA7284’ OR ‘JNJ28431754’
	#3	‘dapagliflozin’ OR ‘farxiga’ OR ‘forxiga’ OR ‘BMS512148’
	#4	‘empagliflozin’ OR ‘jardiance’ OR ‘BI10773’
	#5	‘ipragliflozin’ OR ‘suglat’ OR ‘ASP1941’
	#6	‘luseogliflozin’ OR ‘lusefi’ OR ‘TS071’
	#7	‘remogliflozin’ OR ‘KGT1681’
	#8	‘sotagliflozin’ OR ‘LX4221’
	#9	‘tofogliflozin’ OR ‘apleway’ OR ‘deberza’ OR ‘CSG452’
	#10	‘ertugliflozin’ OR ‘PF04971729’
	#11	#1 OR #2 OR #3 OR #4 OR #5 OR #6 OR #7 OR #8 OR #9 OR #10
**Patient population**	#12	‘heart failure’
	#13	‘chronic kidney disease’ OR ‘renal impairment’ OR ‘renal insufficiency’
	#14	‘diabetes mellitus’
	#15	#12 OR #13 OR #14
**Type of study**	#16	‘randomized’
	#17	‘randomised’
	#18	‘random*’
	#19	#16 OR #17 OR #18
**Combined**	#20	#11 AND #15 AND #19

Appendix Table 3. Risk of bias assessment.

	**Random sequence generation**	**Allocation concealment**	**Blinding of participants and personnel**	**Blinding of outcome assessment**	**Incomplete outcome data**	**Selective reporting**	**Other biases**	**Overall risk of bias**
Bailey et al,[18] 2013	Low	Low	Low	Low	High	Unclear	Unclear	High
Bailey et al,[19] 2015	Unclear	Unclear	Unclear	Low	High	Unclear	Unclear	High
Bode et al,[20] 2015	Low	Low	Unclear	Low	Low	Unclear	Low	Unclear
CANTATA-MSU,[21] 2013	Low	Low	Low	Low	High	Unclear	Low	High
CANVAS Program,[22] 2017	Low	Low	Low	Low	Low	Low	Low	Low
Cefalu et al,[23] 2015	Low	Low	High	Low	High	Unclear	Low	High
CREDENCE,[37] 2019	Low	Low	Low	Low	Low	Low	Low	Low
DAPA-HF,[39] 2019	Low	Low	Low	Low	Low	Low	Low	Low
DECLARE-TIMI 58,[24] 2019	Low	Low	Low	Low	Low	Low	Low	Low
EMPA-HEART CardioLink-6,[25] 2019	Low	Low	Low	Low	Low	Low	Low	Low
EMPA-REG OUTCOME,[26] 2015	Low	Low	Low	Low	Low	Low	Low	Low
EMPA-REG RENAL,[35] 2014	Low	Low	Low	Low	Low	Low	Low	Low

EMPA-RESPONSE-AHF,[38] 2020	Low	Low	Low	Low	Low	Low	Low	Low
Inagaki et al,[27] 2013	Low	Low	Low	Low	High	High	Low	High
Kovacs et al,[30] 2015	Low	Unclear	Unclear	Low	Low	Low	Low	Unclear
Leiter et al,[28] 2014	Low	Low	Low	Low	Low	Unclear	Unclear	Unclear
Mathieu et al,[31] 2015	Low	Low	Unclear	Unclear	Low	Low	Low	Unclear
Rosenstock et al,[29] 2015	Low	Low	Low	Low	Low	Low	Low	Low
Softeland et al,[32] 2017	Unclear	Unclear	Low	Unclear	Low	Low	Low	Unclear
VERTIS RENAL,[36] 2018	Low	Low	Low	Low	Low	Low	Low	Low
Wilding et al,[33] 2012	Low	Low	Low	Low	High	Unclear	Low	High
Yale et al,[34] 2014	Low	Low	Low	Low	Low	Low	Low	Low

Appendix Table 4. Results of sensitivity analyses.

(a) Sensitivity analyses of excluding studies with a high/unclear overall risk of bias, excluding studies with a high/unclear risk of bias in Incomplete outcome data, and using Odds ratio as effective measure

Outcome	Analysis	Result
**AF**	Primary analysis	RR 0.82 (95% CI 0.70 – 0.96)
	Using OR as effect measure	OR 0.82 (95% CI 0.69 – 0.96)
	Excluding studies with high/unclear overall risk of bias	RR 0.82 (95% CI 0.70 – 0.97)
	Excluding studies with high/unclear risk of bias in 'Incomplete outcome data'	RR 0.83 (95% CI 0.70 – 0.96)
**Embolic stroke**	Primary analysis	RR 0.32 (95% CI 0.12 – 0.85)
	Using OR as effect measure	OR 0.32 (95% CI 0.12 – 0.85)
	Excluding studies with high/unclear risk of bias	RR 0.32 (95% CI 0.12 – 0.85)
	Excluding studies with high/unclear risk of bias in 'Incomplete outcome data'	RR 0.32 (95% CI 0.12 – 0.85)
**AFL**	Primary analysis	RR 0.83 (95% CI 0.58 – 1.17)
	Using OR as effect measure	OR 0.83 (95% CI 0.58 – 1.17)
	Excluding studies with high/unclear risk of bias	RR 0.92 (95% CI 0.58 – 1.44)
	Excluding studies with high/unclear risk of bias in 'Incomplete outcome data'	RR 0.87 (95% CI 0.59 – 1.29)
**AF/AFL**	Primary analysis	RR 0.82 (95% CI 0.71 – 0.95)
	Using OR as effect measure	OR 0.82 (95% CI 0.71 – 0.95)
	Excluding studies with high/unclear overall risk of bias	RR 0.82 (95% CI 0.71 – 0.96)
	Excluding studies with high/unclear risk of bias in 'Incomplete outcome data'	RR 0.83 (95% CI 0.72 – 0.96)
**VT**	Primary analysis	RR 0.73 (95% CI 0.53 – 0.99)
	Using OR as effect measure	OR 0.73 (95% CI 0.53 – 0.99)
	Excluding studies with high/unclear risk of bias	RR 0.72 (95% CI 0.53 – 0.98)
	Excluding studies with high/unclear risk of bias in 'Incomplete outcome data'	RR 0.73 (95% CI 0.53 – 0.99)
**Cardiac arrest**	Primary analysis	RR 0.83 (95% CI 0.61 – 1.14)
	Using OR as effect measure	OR 0.83 (95% CI 0.61 – 1.14)
	Excluding studies with high/unclear risk of bias	RR 0.86 (95% CI 0.62 – 1.17)
	Excluding studies with high/unclear risk of bias in 'Incomplete outcome data'	RR 0.86 (95% CI 0.62 – 1.17)

(b) Sensitivity analyses of stratifying trials according to DM versus other baseline conditions

Outcome	Subgroup	Number of trials	Number of participants	RR (95% CI)	P_{hetero}
AF	DM	16	44896	RR 0.84 (95% CI 0.71 – 1.00)	0.39
	Other baseline conditions	5	6297	RR 0.67 (95% CI 0.42 – 1.07)	

Embolic stroke	DM	4	38723	RR 0.32 (95% CI 0.10 – 1.03)	0.96
	Other baseline conditions	2	5482	RR 0.30 (95% CI 0.05 – 1.93)	
AFL	DM	7	36373	RR 0.75 (95% CI 0.52 – 1.09)	0.10
	Other baseline conditions	2	9115	RR 1.96 (95% CI 0.65 – 5.87)	
AF/AFL	DM	17	45818	RR 0.82 (95% CI 0.70 – 0.96)	0.92
	Other baseline conditions	5	6297	RR 0.80 (95% CI 0.52 – 1.23)	
VT	DM	5	34739	RR 0.90 (95% CI 0.56 – 1.42)	0.24
	Other baseline conditions	3	9224	RR 0.62 (95% CI 0.41 – 0.94)	
Cardiac arrest	DM	4	34868	RR 0.74 (95% CI 0.42 – 1.30)	0.82
	Other baseline conditions	3	9883	RR 0.82 (95% CI 0.41 – 1.63)	

Abbreviations used in Table 2: AF, atrial fibrillation; AFL, atrial flutter; VT, ventricular tachycardia; RR, risk ratio; OR, odds ratio; 95% CI, 95% confidence interval; DM, diabetes mellitus; P_{hetero}, P-value for between-subgroup heterogeneity.

Appendix Table 5. GRADE assessment.

A) Atrial fibrillation

Quality assessment							No. of patients		Effect	Quality
No. of studies	Design	Risk of bias	Inconsistency	Indirectness	Imprecision	Other considerations	SGLT2i	Control	Relative risk (95% CI)	
21	Randomized trials	No serious risk of bias[1]	No serious inconsistency	No serious indirectness	No serious imprecision	None	28751	22442	0.82 (0.70 – 0.96)	⊕⊕⊕⊕ High

[1]Although there were some concerns for five trials at high risk of bias (NCT00528879, NCT00528372, NCT01106625, NCT01022112, and NCT00673231), no important difference was noted in sensitivity analysis excluding these studies.

B) Embolic stroke

Quality assessment							No. of patients		Effect	Quality
No. of studies	Design	Risk of bias	Inconsistency	Indirectness	Imprecision	Other considerations	SGLT2i	Control	Relative risk (95% CI)	
6	Randomized trials	No serious risk of bias	No serious inconsistency[1]	No serious indirectness	No serious imprecision	None	24054	20151	0.32 (0.12 – 0.85)	⊕⊕⊕⊕ High

[1]Although numerous SGLT2i agents (canagliflozin, dapagliflozin, and empagliflozin) were evaluated amongst a relatively small number of trials, no significant heterogeneity was observed across trials (p=0.99).

C) Atrial flutter

Quality assessment							No. of patients		Effect	Quality
No. of studies	Design	Risk of bias	Inconsistency	Indirectness	Imprecision	Other considerations	SGLT2i	Control	Relative risk (95% CI)	
9	Randomized trials	No serious risk of bias[1]	No serious inconsistency[2]	No serious indirectness	Serious[3]	None	24801	20677	0.83 (0.58 – 1.17)	⊕⊕⊕O Moderate

[1]Although there were some concerns for the NCT01031680 trial at a high risk of bias and the NCT01210001 trial at an unclear risk of bias, no important difference was noted in sensitivity analysis excluding these studies.

[2]Although numerous SGLT2i agents (canagliflozin, dapagliflozin, and empagliflozin) were evaluated amongst a relatively small number of trials, no significant heterogeneity was observed across trials (p=0.45).

[3] Imprecision was downgraded because the 95% of the relative risk was sufficiently wide that the estimate could include appreciable harm or benefit of the intervention (thresholds: 0.75 and 1.25).

D) Atrial fibrillation/Atrial flutter

Quality assessment							No. of patients		Effect	Quality
No. of studies	Design	Risk of bias	Inconsistency	Indirectness	Imprecision	Other considerations	SGLT2i	Control	Relative risk (95% CI)	
22	Randomized trials	No serious risk of bias[1]	No serious inconsistency	No serious indirectness	No serious imprecision	None	29211	22904	0.82 (0.71 – 0.95)	⊕⊕⊕⊕ High

[1]Although there were some concerns for six trials at high risk of bias (NCT00528879, NCT00528372, NCT01106625, NCT01031680, NCT01022112, and NCT00673231) and five trials at an uncertain risk of bias (NCT01106651, NCT01210001, NCT01042977, NCT01646320, and NCT01734785), no important difference was noted in sensitivity analysis excluding these studies.

E) Ventricular tachycardia

Quality assessment							No. of patients		Effect	Quality
No. of studies	Design	Risk of bias	Inconsistency	Indirectness	Imprecision	Other considerations	SGLT2i	Control	Relative risk (95% CI)	
8	Randomized trials	No serious risk of bias[1]	No serious inconsistency[2]	No serious indirectness	No serious imprecision	None	23884	20079	0.73 (0.53 – 0.99)	⊕⊕⊕⊕ High

[1]Although the NCT03200860 trial had an unclear risk of bias, it contributed little to the overall treatment effect estimate.

[2]Although numerous SGLT2i agents (canagliflozin, dapagliflozin, and empagliflozin) were evaluated amongst a relatively small number of trials, no significant heterogeneity was observed across trials (p=0.61).

F) Cardiac arrest

Quality assessment							No. of patients		Effect	Quality
No. of studies	Design	Risk of bias	Inconsistency	Indirectness	Imprecision	Other considerations	SGLT2i	Control	Relative risk (95% CI)	
7	Randomized trials	No serious risk of bias[1]	No serious inconsistency[2]	No serious indirectness	Serious[3]	None	24463	20288	0.83 (0.61 – 1.14)	⊕⊕⊕O Moderate

[1]Although there were some concerns for the NCT00528879 trial with a high risk of bias, it contributed little to the overall treatment effect estimate.

[2]Although numerous SGLT2i agents (canagliflozin, dapagliflozin, and empagliflozin) were evaluated amongst a relatively small number of trials, no significant heterogeneity was observed across trials (p=0.44).

[3]Imprecision was downgraded because the 95% of the relative risk was sufficiently wide that the estimate could include appreciable harm or benefit of the intervention (thresholds: 0.75 and 1.25).
